# Impact of a High-Fat Diet at a Young Age on Wound Healing in Mice

**DOI:** 10.3390/ijms242417299

**Published:** 2023-12-09

**Authors:** Kevin Arnke, Pablo Pfister, Gregory Reid, Mauro Vasella, Tim Ruhl, Ann-Kathrin Seitz, Nicole Lindenblatt, Paolo Cinelli, Bong-Sung Kim

**Affiliations:** 1 Department of Plastic Surgery and Hand Surgery, Burn Center, University Hospital Zurich, 8006 Zurich, Switzerland; kevin.arnke@usz.ch (K.A.); ann-kathrin.seitz@usz.ch (A.-K.S.); nicole.lindenblatt@usz.ch (N.L.); bong-sung.kim@usz.ch (B.-S.K.); 2 Center for Surgical Research, University Hospital Zurich, University of Zurich, 8006 Zurich, Switzerland; paolo.cinelli@usz.ch; 3 Department of Surgery, Triemli City Hospital Zurich, 8063 Zurich, Switzerland; 4 Department of Plastic Surgery, Hand Surgery-Burn Center, University Hospital RWTH Aachen, 52074 Aachen, Germany; truhl@ukaachen.de; 5 Department of Trauma Surgery, University Hospital Zurich, 8006 Zurich, Switzerland

**Keywords:** obesity, wound healing, high-fat diet, mice, young age

## Abstract

As the prevalence of juvenile-onset obesity rises globally, the multitude of related health consequences gain significant importance. In this context, obesity is associated with impaired cutaneous wound healing. In experimental settings, mice are the most frequently used model for investigating the effect of high-fat diet (HFD) chow on wound healing in wild-type or genetically manipulated animals, e.g., diabetic *ob*/*ob* and *db*/*db* mice. However, these studies have mainly been performed on adult animals. Thus, in the present study, we introduced a mouse model for a juvenile onset of obesity. We exposed 4-week-old mice to an investigational feeding period of 9 weeks with an HFD compared to a regular diet (RD). At a mouse age of 13 weeks, we performed excisional and incisional wounding and measured the healing rate. Wound healing was examined by serial photographs with daily wound size measurements of the excisional wounds. Histology from incisional wounds was performed to quantify granulation tissue (thickness, quality) and angiogenesis (number of blood vessels per mm^2^). The expression of extracellular matrix proteins (collagen types I/III/IV, fibronectin 1, elastin), inflammatory cytokines (MIF, MIF-2, IL-6, TNF-α), myofibroblast differentiation (α-SMA) and macrophage polarization (CD11c, CD301b) in the incisional wounds were evaluated by RT-qPCR and by immunohistochemistry. There was a marked delay of wound closure in the HFD group with a decrease in granulation tissue quality and thickness. Additionally, inflammatory cytokines (MIF, IL-6, TNF-α) were significantly up-regulated in HFD- when compared to RD-fed mice measured at day 3. By contrast, MIF-2 and blood vessel expression were significantly reduced in the HFD animals, starting at day 1. No significant changes were observed in macrophage polarization, collagen expression, and levels of TGF-β1 and PDGF-A. Our findings support that an early exposition to HFD resulted in juvenile obesity in mice with impaired wound repair mechanisms, which may be used as a murine model for obesity-related studies in the future.

## 1. Introduction

The global surge of obesity across all population ages and genders fuels major diseases, including atherosclerosis, type 2 diabetes mellitus, and cancer [1]. In surgical patients, obesity has been linked to higher incidences of fascial dehiscence, wound infections, and mortality [2]. Moreover, one crucial aspect of obesity is its negative impact on cutaneous wound healing [2]. People with obesity bear higher risks for impaired cutaneous wound healing and total wound failure [2]. These detrimental effects gain importance as the prevalence of obesity reaches alarming levels among young individuals: according to recent data, 13.9% of 2–5-year-old children and 18.4% of school-aged children in the United States exhibit an excessively high body mass index (BMI) [3]. Importantly, these children are not only more likely to become obese in adulthood, as approximately 80% remain obese but are also more prone to a myriad of serious health threats [4].

For a physiologic wound healing process, a complex interaction among various cellular and soluble factors of the skin is involved, which leads to the well-known progression of four wound healing phases: coagulation and hemostasis, inflammation, proliferation, and remodeling [5,6]. Throughout all phases, fibroblasts carry a central role in orchestrating physiological wound repair [7]. During the temporal progression of the wound healing process, resident fibroblasts proliferate and create contractile granulation tissue, facilitating wound closure with the differentiation into myofibroblasts [7,8]. These myofibroblasts act as activated repair cells, which are mainly responsible for the production of a newly formed extracellular matrix in response to a repair stimulus [9]. In addition, stromal vascular cells in the subcutaneous tissue promote wound repair through the release of growth factors and cytokines that consequently interact with inflammatory cells that are recruited to the injured area [6]. These inflammatory cells are of particular interest, as they can negatively impact wound healing outcomes in conditions like type 2 diabetes and obesity [6]. In this context, increased degranulation of mast cells has been identified as a crucial contributor to the deranged repair mechanism in diabetes mellitus [10]. However, this contribution has yet to be verified in obesity-related wound healing [11]. In obesity, the impairment of wound healing has been linked to an abnormal inflammatory phase that involves a state of heightened inflammation, both systemically and in local adipose tissue [12]. Several elevated pro-inflammatory chemokines and cytokines have been recognized as contributing factors in obese wound-healing animal models, such as tumor necrosis factor α (TNF-α), migration inhibitory factor (MIF), and interleukin-6 (IL-6) [13,14,15]. In excisional wounds, MIF has been shown to slow down wound healing through an increase in inflammatory cytokines, such as TNF-α, released upon stimulation of MIF by M1 macrophages [16]. A significant portion of our understanding of these processes has been derived from studies conducted on mice [17]. A popular model used in obesity research is the use of wild-type (wt) mice with a high-fat diet (HFD) driven obese phenotype [18,19]. In this model, the investigational feeding start has commonly been at an adult mouse age of 6 weeks [18,19]. Other common models used are the leptin receptor-deficient diabetes (*db*/*db*) strains and leptin-deficient obese (*ob*/*ob*) strains [20,21,22]. Both strains were used in experiments for cutaneous wound healing early on [23,24]. In contrast to the HFD model, the impaired glucose metabolism (and not obesity) is the major phenotype in the *db*/*db* and *ob*/*ob* strains and thus gives a more comprehensive insight into pathomechanisms related to diabetes and its complications.

Considering that obesity often begins at an early age in humans due to an HFD, our goal was to establish a mouse model for studying juvenile-onset obesity. Thus, we exposed C57BL/6 wild-type mice to an HFD at a relatively early age of 4 weeks and compared them to C57BL/6 wild-type mice receiving a regular diet (RD). This translates to a young human age just before puberty sets in, usually occurring around day 42 [25]. Subsequently, we aimed to examine the impacts of this model on both incisional and excisional wound healing processes.

## 2. Results

### 2.1. Early High-Fat Diet Induces Obese Phenotype and Delays Normal Wound Healing

**Figure 1:**
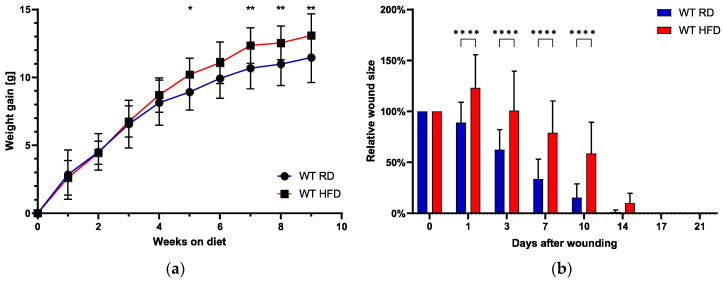
Mean weight gain in grams (g) graphed weekly from investigational feeding week 1 to week 9 (**a**) comparing the RD and HFD group. Relative wound size in relation to the initial wound (100%) over time in days after the wound until day 21 (**b**). Bars indicate the mean ± SEM obtained from 6 wounds. * *p* < 0.05. ** *p* < 0.01. **** *p* < 0.0001.

We measured the animal’s weight gain weekly for an overall duration of 9 weeks (Figure 1a). There was a statistically significant increase in weight gain in the HFD group, starting at 5 weeks after the onset of the investigational feeding. The increase in weight gain was also significant for the time points 7, 8, and 9 weeks after feeding initiation in the HFD group.

After the feeding period of 9 weeks, wounding was performed simultaneously in both groups (HFD/RD mice). The relative size of the excisional wound, with an initial size of 6 cm^2^, was assessed daily until full wound closure, which occurred until day 14 in most animals, irrespective of the group (Figure 1b). Overall, we observed a trend towards earlier total wound closure in RD-fed mice, although this effect was not statistically significant.

**Figure 2:**
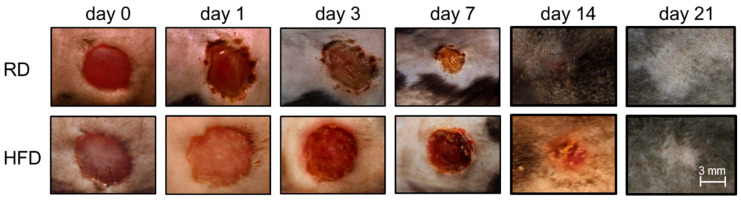
Photographic documentation sample of excision wounds in the RD and HFD group from the day of wound (day 0) until day 21.

When looking at acute wound closure, represented by days 1–10 after wounding, we observed a statistically highly significant delay in mice with an obese phenotype (HFD-fed mice). The mean relative wound size one day after wounding significantly increased to almost 125% of the original wounding area in the obese phenotype. Relative wound sizes remained significantly increased until 10 days after the initial wounding. In contrast, the RD-fed mice showed a steady decrease starting at day 1 with 89.00% (±20.01%). The relative wound size in the HFD group started decreasing with a marked delay after day 3. Exemplary photographic documentation (Figure 2) visualizes the graphed data.

### 2.2. Granulation Tissue Quality and Thickness Impaired with an Early High-Fat Diet

We evaluated the thickness of the granulation tissue in the wounded areas, stained with TCG, and compared it to the thickness of the healthy control tissue on the non-injured portion of the histology slide. Following that, we evaluated the quality of the granulation tissue in accordance with the description provided in Section 4.7.

**Figure 3:**
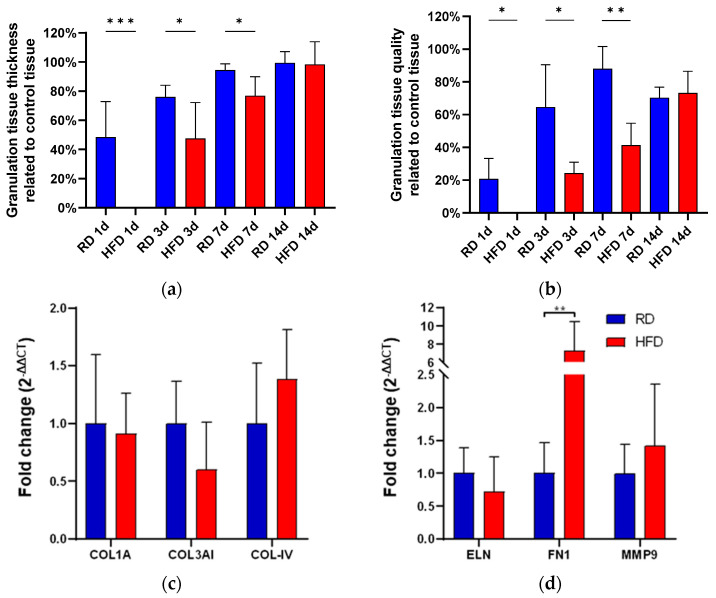
Granulation tissue thickness (**a**) and granulation tissue quality (**b**) comparing the RD and HFD group from day 1, day 3, day 7, and day 14 after wounding. Relative mRNA fold change (RT-qPCR) of collagen types 1A, 3AI, and 4 (**c**) at day 3 after wounding. Relative mRNA fold change (RTqPCR) of elastin (ELN), fibronectin 1 (FN1), and matrix-metalloproteinase 9 (MMP9) at day 3 after wounding (**d**). Bars indicate the mean ± SEM obtained from 6 wounds. * *p* < 0.05. ** *p* < 0.01. *** *p* < 0.001.

The assessment of granulation thickness showed significant differences: one day after the injury, there was no granulation tissue formation at all in the HFD group, compared to 48.45% granulation tissue thickness in the RD group (*p* < 0.001, Figure 3a). We noted analogous findings at subsequent time points, day 3 and day 7, where the obese phenotype exhibited significantly reduced granulation tissue thickness. Looking at granulation tissue quality, we found similar results. Granulation tissue quality was significantly reduced on day 1, 3, and day 7 post-injury (Figure 3b). Two weeks after the injury, granulation tissue thickness and quality reached comparable levels.

Gene expression levels of collagen types I, III, and IV were analyzed in both groups (RT-qPCR). Even though no statistical significance was observed, collagen type III showed a tendency to higher relative expression in the regular diet group (*p* = 0.090), while collagen type IV was slightly higher in the HFD group (*p* = 0.090, Figure 3c). The expression of fibronectin 1 was 8× higher in the HFD-fed mice in comparison with the control group (*p* < 0.01, Figure 3d). Elastin and metalloproteinase 9 mRNA expression showed no statistically significant difference (Figure 3d).

### 2.3. Delayed Myofibroblast Appearance in HFD-Fed Mice

**Figure 4:**
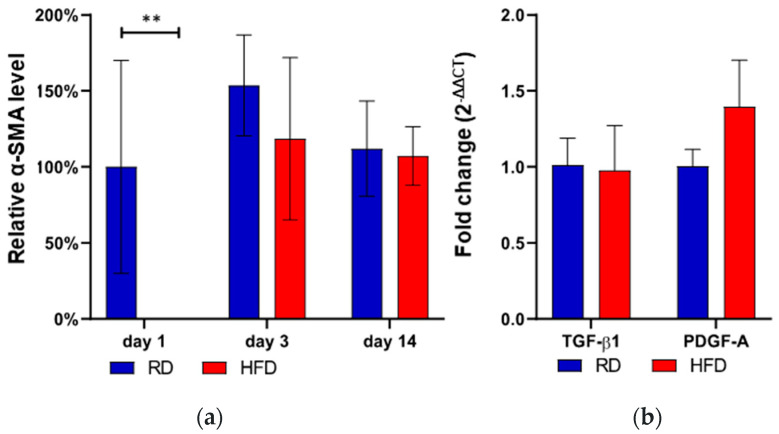
α-SMA protein expression (IHC, α-SMA-Ab) at day 1, day 3, and day 14 after wounding comparing the RD and HFD group (**a**). The α-SMA protein expression was normed to regular diet (RD) mice on the first day after injury. Relative mRNA fold change (RT-qPCR) of TGF-β1 and PDGF-A at day 3 after wounding (**b**). Bars indicate the mean ± SEM obtained from 6 wounds. ** *p* < 0.01.

Subsequently, we examined the α-SMA protein expression as a marker for myofibroblast appearance [26]. One day after the injury, we did not detect any α-SMA protein expression in HFD-fed mice, as compared to the substantiation in the RD-fed group (*p* < 0.01, Figure 4a). This was measured by means of α-SMA staining quantification (IHC). The detection of α-SMA protein expression in HFD-fed mice started at day 3 after wounding. For the time points day 3 and day 14, there was a slight trend towards a reduced expression in the obese phenotype without reaching statistical significance.

In the next step, we assessed the expression levels of PDGF-A and TGF-β1 through RT-qPCR analysis, both of which are known to upstream regulate phenotypic changes in fibroblasts, converting them into myofibroblasts [27]. Three days after the injury, there were no statistically significant variations in the mRNA expressions of both groups, though there was a tendency for higher PDGF-A expression in the HFD-fed animals (Figure 4b).

### 2.4. Early High-Fat Diet Increases Mediators of Inflammation

**Figure 5:**
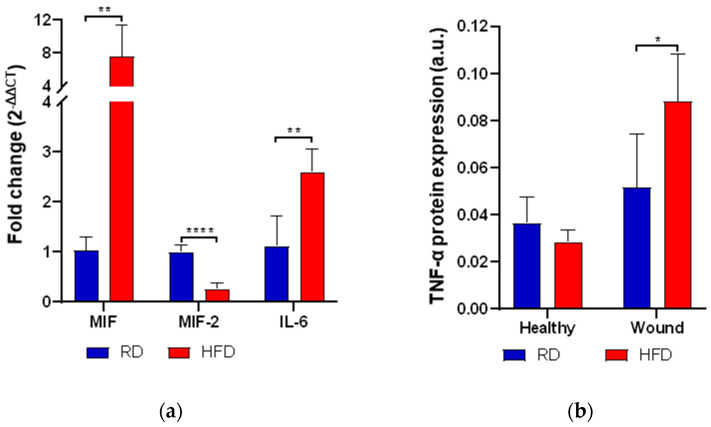
Relative mRNA fold change (RT-qPCR) of MIF, MIF-2, and IL-6 compared to the HFD group on day 3 after wounding (**a**). TNF-α protein expression (IHC, TNF-α-Ab) comparing healthy tissue to wounded tissue at day 3 after wounding (**b**). Bars indicate the mean ± SEM obtained from 6 wounds. * *p* < 0.05. ** *p* < 0.01. **** *p* < 0.0001.

Next, we assessed the expression (RT-qPCR) of various pro- and anti-inflammatory markers such as TNF-α, IL-6 macrophage migration inhibitory factor (MIF), and its homolog D-dopachrome tautomerase (D-DT, also called MIF-2). On the third day post-wounding, the measured inflammatory markers were significantly up-regulated in the HFD-fed group. We observed an over sixfold increase in MIF mRNA expression in mice on a high-fat diet (*p* < 0.01, Figure 5a). In addition, the levels of TNF-α protein expression were significantly elevated in these obese mice, nearly doubling those observed in lean mice (*p* < 0.05, Figure 5b). Moreover, the expression of IL-6 mRNA levels was more than twice as high in the obese animals (*p* < 0.05, Figure 5a). By contrast, the measured anti-inflammatory marker, MIF-2 mRNA, was significantly reduced by more than fourfold in the obese animals (*p* < 0.0001, Figure 5a).

### 2.5. Macrophage Quantification and M1/M2 Phenotype Assessment

**Figure 6:**
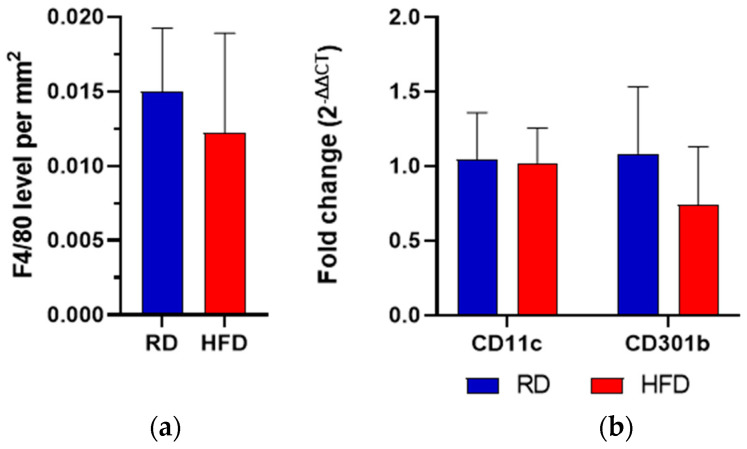
F4/80 level expression per mm^2^ (IHC, F4/80-Ab) at day 3 after wounding (**a**). Relative mRNA fold change (RT-qPCR) of CD11c and CD301b comparing the RD to the HFD group at day 3 after wounding (**b**). Bars indicate the mean ± SEM obtained from 6 wounds.

To histologically quantify the macrophages in the investigated wound area, the F4/80 molecule was targeted in immunohistochemistry [28]. No significant difference was observed comparing F4/80 levels per mm^2^ wound area in both groups three days after injury (Figure 6a). Following that, we wanted to detect the macrophage M1/M2 phenotype, using CD11c as an M1 marker and CD301b as an M2 marker.

In both groups, CD11c and CD301b mRNA exhibited comparable levels after skin injury. The HFD group showed a trend towards lower CD301b expression, although this difference was not statistically significant (Figure 6b).

### 2.6. Early Vascularization Is Decreased after Wounding in Early HFD-Fed Animals

**Figure 7:**
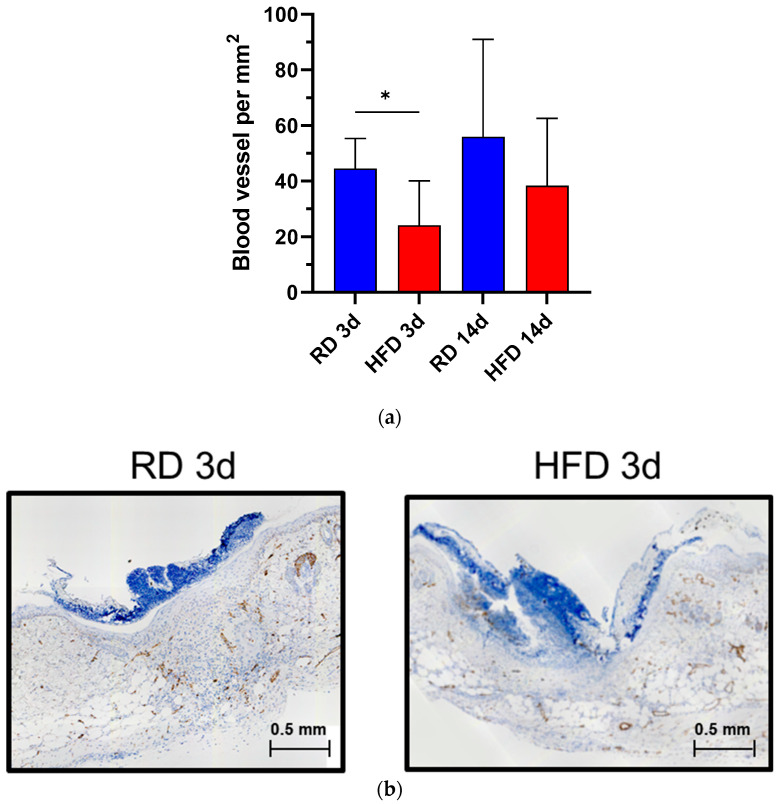
Blood vessel expression per mm^2^ comparing the RD and HFD group at day 3 and day 14 after wounding (**a**). Bars indicate the mean ± SEM obtained from 6 wounds. Example of immunohistochemistry staining for blood vessels on day 3 after wounding (**b**), (IH.C). The blue color represents CD 31-Ab positive cells. * *p* < 0.05.

Lastly, we wanted to investigate the neo-vascularization of the de novo tissue. The blood vessels were individually counted according to their distinct histological morphology as an indicator of vascularization of the wounds. Blood vessels per mm^2^ in the wounded area were significantly reduced by almost half in the obese phenotype (HFD) three days after injury (*p* < 0.05, Figure 7a). Fourteen days after injury, blood vessels showed similar levels, with a trend towards a reduced expression in the HFD group. An example of immunohistochemistry is shown in Figure 7b.

## 3. Discussion

Obesity remains a fundamental healthcare issue, and the understanding of its pathophysiological consequences is an imperative focus in current research investigations [2]. With the rising number of individuals with obesity, health threats, including the disruption of cutaneous wound repair, gain significant importance [1]. Understanding these pathologic processes in the mouse model has generated interest to extend these findings to benefit human patients. Previous basic research papers in murine studies have focused mainly on the diabetic *ob*/*ob* and *db*/*db* mouse models for the examination of impaired wound healing [23,24]. Seitz and colleagues conducted probably the most detailed comparison of wound healing in wild-type mice between a normal diet and HFD so far, which resulted in a general delay in wound closure in the obese phenotype [29]. Furthermore, they measured HFD-induced impairments in wound inflammation, reepithelization, angiogenesis, and wound contraction [29]. In contrast to our study, the authors started the HFD at an adult mouse age of 6 weeks in the C57Bl/6J group (and at 12 weeks for the C57Bl/6J-*ob*/*ob* group). Their investigation was performed in line with most murine studies on wound repair, starting the diet at the age of 6 weeks or more [18,19,30,31].

In our experiment, we started the investigational feeding period at 4 weeks of mouse age, which relates to a juvenile human age group [25]. We observed a significant increase in body weight, beginning after 5 weeks of diet, and successfully induced an obese phenotype in the C57BL/6 mice after a total of 9 weeks of continuous feeding with a high-fat diet. Of note, the onset of obesity was prolonged in our model, as compared to the literature, with a usual HFD initiation at 6 weeks [32]. Nevertheless, a significant increase in the C57BL/6 mice’s body weight was measurable. This increase represents our main finding, as implementing an early HFD has not been described in the previously published literature. The magnitude of weight gain cannot be compared to the steep weight gain curve of a genetically modified mouse model, like the *ob*/*ob* strain, but the negative effects on wound healing are clearly demonstrated in our experiment.

The early exposure to an HFD resulted in a significant delay of granulation tissue formation and a significant delay of acute wound closure up to ten days after wounding. In addition to the prolonged onset of granulation tissue formation, the *de novo* tissue quality was significantly reduced in the obese phenotype. This was further detailed by analyzing the mRNA expression of fibronectin and collagen type III mRNA, both of which are important components of this initial provisional matrix [33]. We observed a significant and nearly eightfold increase in mRNA expression of fibronectin 1 three days after injury. In the context of wound healing, excess levels of fibronectin have been associated with abnormally healing wounds and fibrosis [34]. A key role of its activity is during the beginning of the proliferation stage, in which it forms a scaffold for myofibroblasts and collagen deposits as part of the newly formed granulation tissue [35,36]. This production process is balanced, and any changes in this equilibrium can lead to unfavorable effects on wound healing [34]. Based on our observations, it appears that the initial granulation tissue in the HFD group is predominantly composed of fibronectin. This, in turn, could serve as an important marker for the disrupted repair mechanism in the obese phenotype mice. Consequently, we examined the upstream regulator of fibronectin expression, PDGF-A, but found no significant difference. Although there was a trend towards a higher PDGF-A expression, this does not allow for an association. The conclusive explanation for the increase in fibronectin thus remains uncertain. Either our analysis did not reveal the involvement of PDGF-A in this pathway, or the primary producers of fibronectin, known as “wound fibroblasts”, were increased in quantity [37,38].

Overall, we cannot demonstrate causality in this experiment, but our additional data points reveal a certain insight into the involved wound healing markers, each as part of the different phases of wound healing, including the hemostasis/inflammation phase, proliferation phase, and remodeling phase [39,40]. Therefore, three findings are emphasized here.

The early exposure to an HFD led to a significant alteration of cytokine levels. The expression of MIF mRNA in HFD-fed mice after skin injury was significantly increased, which has been suggested to promote inflammation in wound repair by up-regulating pro-inflammatory cytokines such as TNF-α [16]. Since MIF has been described as an upstream regulator of TNF-α, the observed increase in TNF-α protein supports this previously reported connection [41]. In line with previous reports that suggest an inhibitory role of MIF in wound repair, this could play an important part in the pathologic healing pathway in the obesity-induced phenotype [42,43]. Conversely, the anti-inflammatory cytokine MIF-2 showed a significant decrease. Although the precise interaction of the MIF superfamily members (MIF and MIF-2) is not yet fully understood, they appear to have reciprocal roles in wound healing: MIF as a potent pro-inflammatory chemokine and MIF-2 as a facilitator of dermal repair, both through fibroblast interaction [44]. In our wound healing model, the introduction of an obese phenotype appears to enhance the pro-inflammatory balance through the MIF superfamily axis.

Since MIF and MIF-2 are both released through activated macrophages, we wanted to elucidate their role and their respective polarization in our experiment, quantifying the number of macrophages in general through the F4/80 molecule and using CD11c as an M1- and CD301b as a M2-marker [45,46]. In general, M1 macrophages are considered to play pro-inflammatory roles, while M2 macrophages are specialized in tissue repair [47]. Macrophages, as part of the myeloid cell line, are induced to M1 or M2 phenotypes according to the external stimuli [47]. We observed a trend towards a decreased expression of CD301b mRNA in the HFD group, although this change did not reach statistical significance. A reduction of CD301b mRNA could indicate a deficient transition of pro-inflammatory M1 macrophages to pro-healing M2 macrophages, which would consequently be associated with defective wound closure and poor angiogenesis [48]. A more elaborate analysis of macrophage polarization markers, including an in-depth analysis of M2 subtypes, may hold future relevance in unraveling a possible effect on phenotype polarization in obesity-related wound healing disorders.

At last, we assessed the α-SMA protein as a marker for myofibroblast differentiation. We did not detect α-SMA protein expression one day after the injury, which we considered to be due to a failed early myofibroblast differentiation. From the literature, the appearance of myofibroblasts is considered an indicator of wound healing and contraction [26,49]. Subsequently, failed myofibroblast differentiation results in adverse effects on wound healing, particularly in terms of tissue contraction [26,49,50]. This finding aligns with the significant delay in the closure of wounds we observed during the initial 10-day period. The primary source of myofibroblasts is local fibroblastic progenitors, which are activated by cytokines and growth factors [51,52,53]. Among these, TGF-β1 and PDGF-A regulate the fibroblast-to-myofibroblast transition (FMT) in wound healing [51]. Although PDGF-A was sufficiently expressed in the HFD group, the FMT was still insufficient, pointing to a failed recruitment of fibroblastic progenitors as a possible cause.

Our study does not come without limitations. The study was primarily designed to introduce an early HFD to create a juvenile-onset obesity wound healing model. Although we successfully induced an obese phenotype and demonstrated the impact on wound healing, the translation to human juvenile-onset obesity remains to be confirmed. In addition, we tried to minimize sample size and thus could have missed statistically significant differences. As a limitation to the specification of macrophage polarization, CD11c, and CD301b were measured in pooled wound tissue, which is less accurate than flow cytometry as it does not distinguish between the different cell fractions within the tissue. Moreover, a more in depth analysis of the wound healing processes with additional methodological approaches and the use of a regular-onset obesity control-group could have provided a more elaborate analysis. In the next step, a direct comparison of wound healing in a regular-onset and juvenile-onset obesity phenotype would be of interest.

## 4. Materials and Methods

### 4.1. Ethical Approval

In vivo, experiments were approved by the Cantonal Veterinary Office, Zurich (ZH004/19) and were in accordance with the Swiss Animal Protection Law and the European Directive 2010/63/EU of the European Parliament and of the Council on the Protection of Animals used for Scientific Purposes.

### 4.2. Animals

A total of 30 C57BL/6 wild-type male mice were used for mouse experiments, purchased from Charles River Laboratories (Sulzfeld, Germany). The animals were housed under a regular 12h:12h light:dark cycle.

### 4.3. Diets

We generated a diet-induced obesity (DIO) model to mimic the development and the state of obesity. For the DIO, we used chows consisting of 60 kJ% fat (ssnif Spezialdiäten GmbH, Soest, Germany). Mice were 4 weeks old when the feeding was initiated, with an overall feeding period of 9 weeks and weekly weighing. The animals of the control group received a regular diet (RD) consisting of 4.5 kJ% fat (Kliba Nafag, Kaiseraugst, Switzerland). Food and water access was set *ad libitum*.

### 4.4. Wounding of Mice

**Figure 8:**
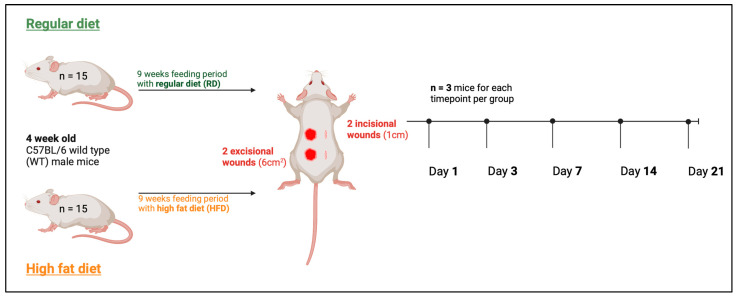
Visualization of the experimental design.

Figure 8 shows the experimental setup. The wounding was performed after a feeding period of 9 weeks when mice were 13 weeks old. Two incisional wounds (1 cm) and two excisional wounds (6 cm^2^) were set at the dorsum of each experimental animal. For the incision wound, the dorsum was incised down to the panniculus carnosus with a 15-blade scalpel. For excision wounds, the skin and the panniculus carnosus muscle were completely removed and not further splinted. We used incisional and excisional wounding based on Ansell et al. [54]. Briefly described, two-incision wounds were set caudally and cranially on the left side with a scalpel, and two excision wounds were placed caudally and cranially on the right side of the back by using a 6 mm^2^ biopsy puncher. The wounds include the epidermis, dermis, hypodermis, and the panniculus carnosus muscle. In accordance with the “reduction” dimension of the 3R principles in animal experimentation, we used two wounds per incisional/excisional type on each mouse, which allowed us to halve the number of animals as each wound counted as an individual experiment [55]. A subcutaneous buprenorphine injection (0.1 mg per kg body weight) was applied before wounding. Mice were anesthetized with 5% isoflurane for initiation, with a reduction to around 3–5% during the intervention. The back of each mouse was shaved and disinfected using 70% EtOH. To avoid hypothermia the intervention was carried out on a heating mat (37 °C). Postoperatively, the mice received an additional postoperative buprenorphine injection and subsequent access to oral buprenorphine via the drinking water.

To assess the healing process (macroscopic analysis), photographic documentation was performed using a camera fixator to ensure reproducibility by establishing a standardized distance from the camera to the wound. Time points for sample collection were 1, 3, 7, 14, and 21 days after wounding. Only for photographic wound documentation, additional time points were implemented on day 10 and day 17. For each sample collection time point, three mice (n = 6 wounds) for the RD and three mice (n = 6 wounds) for the HFD group were euthanized by CO_2_ inhalation. After euthanasia, the wound beds, including the surrounding tissue, were completely excised for further analysis. Wound size was analyzed using Photoshop (Adobe Inc., Mountain View, CA, USA) and ImageJ (National Institutes of Health, Bethesda, MD, USA) for pixel count.

### 4.5. Real-Time Quantitative PCR (RT-qPCR)

RNA was isolated from frozen tissue, which had been homogenized in 1 mL TRIzol^®^ for 3 min, according to a previously reported method by Chomczynski et al. [56]. The cDNA was formed by using the QuantiTect Reverse Transcription Kit (QIAGEN AG, Hilden, Germany) and 500 ng of total RNA. The cDNA was diluted 1:10 with RNase-free water. RT-qPCR was performed using 2 µL diluted cDNA and the Rotor-Gene SYBR Green PCR Kit (QIAGEN AG, Hilden, Germany) according to the instruction manual. The analyzed genes and their corresponding primers are listed in Table 1. Gene expression was normalized on the two housekeeping genes, *Rps29* and *Actab*, according to the method from Taylor et al. [57].

### 4.6. Histology: Masson–Goldner-Trichrome Staining and Immunohistochemistry

The tissue was fixed in 4% paraformaldehyde for histological analysis. Masson–Goldner’s trichrome (TCG) staining was used for collagen deposit and granulation tissue development [58]. Immunohistochemistry (IHC) antibodies (unless otherwise specified, all antibodies were purchased from Abcam, Cambridge, UK) were visualized by using a secondary antibody conjugated to horseradish peroxidase and [3,3]’-diaminobenzidine (DAB) solution. The antibodies used for IHC staining are listed in Table 2, and examples are shown in Supplementary Figure S1. For the quantification of staining, we used ImageJ (with the color deconvolution technique and optical density (OD) calculation as follows: OD=LOG10 (max grey value/mean grey value). The color deconvolution DAB was used as proposed by the program. The color deconvolution for Masson–Goldner’s trichrome (TCG) staining self-established color vectors using healthy tissue of the same animal.

### 4.7. Granulation Tissue Quality Assessment

Collagen fibers represent the majority of the extracellular matrix in cutaneous connective tissue [59]. Therefore, we quantified the collagen deposition of the newly formed granulation tissue and compared it with the collagen deposition of uninjured skin within the same mouse. This enabled us to make a statement about the quality of the granulation tissue.

Collagen deposition in the different tissues was quantified as follows: at first, the color vector for the turquoise-stained collagen fibers in the dermis was determined by using healthy skin tissue of five different unwounded lean mice. The determined vector values were used for color deconvolution to split the picture into separated color channels. Afterward, the OD was examined in five different regions in the wound area and as a reference in healthy tissue of the same mouse using a defined region of interest (ROI). The average of the five wound area values was calculated and set in relation to the mean value of the healthy tissue.

### 4.8. Statistical Analysis

Statistical analysis was performed using GraphPad Prism Version 8.0 (GraphPad Software Inc., Boston, MA, USA). Data analysis was carried out using a two-way analysis of variance (ANOVA), unpaired two-tailed *t*-test, and Tukey’s post-hoc test. Each wound was counted as an individual n. All data are presented as mean ± standard error of the mean (± SEM). Statistical significance was set at *p*-values < 0.05.

## 5. Conclusions

Conclusively, in accordance with previous findings, exposure to an early HFD leads to an impairment of cutaneous wound healing mechanisms in mice after skin injury. While we observed a delayed emergence of myofibroblasts in wounds of HFD mice, the underlying involvement of other crucial cell fractions, including mast cells in the obesity progression and wound healing remains subject to further analysis. Our observation supports the use of an early exposition of HFD as a mouse model for juvenile-onset obesity, giving researchers the advantage of shorter waiting periods and providing insight into the obesity-related impact on wound healing.

## Figures and Tables

**Table 1:** Primers used in the experiment listed alphabetically.

Gene Name	Forward Primer (5′-3′)	Reverse Primer (5′-3′)
*CD11c*	TGC CAG GAT GAC CTT AGT GTC G	CAG AGT GAC TGT GGT TCC GTA G
*CD301b*	GAC TGA GTT CTC GCC TCT GG	CTG GGA AGG AAT TAG AGC AAA CT
*Col I*	CTG GCG GTT CAG GTC CAA TG	GAA GCC TCG GTG TCC CTT CA
*Col III*	GAC CAA AAG GTG ATG CTG GAC AG	CAA GAC CTC GTG CTC CAG TTA G
*Col IV*	GGT GTG CGG TTT GTG AAG CA	TGG CGT GGG CTT CTT GAA CA
*Eln*	TCC TGG GAT TGG AGG CAT TGC A	ACC AGG CAC TAA ACC TCC AGC A
*Fn*	CGG ACG CTG CGA AAA GAT GA	ACT TGG CTG GCA ACC CTT CT
*Il-6*	TAC CAC TTC ACA AGT CGG AGG C	CTG CAA GTG CAT CAT CGT TGT TC
*Mif*	CGC TTT GTA CCG TCC T	CGT GCC GCT AAA AGT CA
*Mif-2*/*D-Dt*	CTC TTC TCC CGC TAA CAT GC	TCA TGC CAG GTC GTA TCG TA
*Pdgf A*	GCA AGA CCA GGA CGG TCA TTT AC	TGT TCA GGA ATG TCA CAC GCC
*Tgf-β1*	TGA TAC GCC TGA GTG GCT GTC T	CAC AAG AGC AGT GAG CGC TGA A

**Table 2:** Antibodies used for immunohistochemical staining.

Primary Antibody	Host Species	Used Dilution	Secondary Antibody-HRP
CD31	rabbit (polyclonal)	1:50	anti-rabbit
F4/80	rat IgG (monoclonal)	1:400	anti-rat IgG
F(ab)	goat IgG (polyclonal)	1:50	-
α-SMA	mouse (monoclonal)	1:2	anti-mouse
tumor necrosis factor-α	rabbit (monoclonal)	1:100	anti-rabbit

## Data Availability

The data presented in this study are available on request from the corresponding author.

## Supplementary Materials

The following supporting information can be downloaded at https://www.mdpi.com/article/10.3390/ijms242417299/s1.
