# Supplementary material for: Impact of a High-Fat Diet at a Young Age on Wound Healing in Mice
Source: Int J Mol Sci. 2023 Dec 9;24(24):17299. doi: 10.3390/ijms242417299 (PMC10743676; doi:10.3390/ijms242417299)
Supplement: Supplementary file 1 [file ijms-24-17299-s001.zip › ijms-2744737-supplementary.pdf]

Supplementary Figure S1

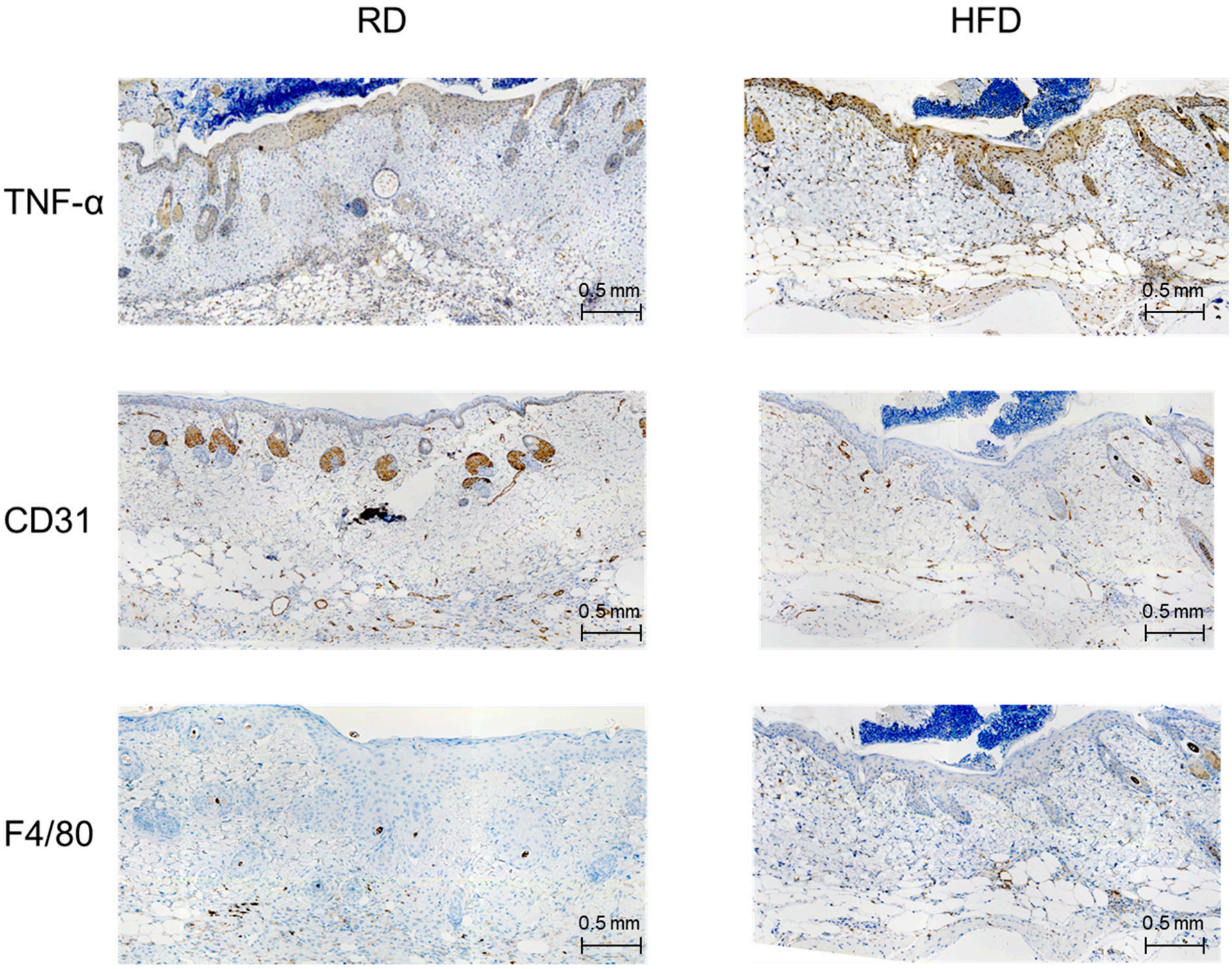

Supplementary Figure S1: Representative images of immunohistochemical expression of TNF-α, CD 31 and F4/80 in the high fat diet (HFD) and regular diet (RD) group.
